# The Synergy between Bio-Aggregates and Industrial Waste in a Sustainable Cement Based Composite

**DOI:** 10.3390/ma14206158

**Published:** 2021-10-17

**Authors:** Cătălina Mihaela Grădinaru, Adrian Alexandru Șerbănoiu, Radu Muntean, Bogdan Vasile Șerbănoiu

**Affiliations:** 1 Faculty of Civil Engineering and Building Services, “Gheorghe Asachi” Technical University of Iași, 700050 Iași, Romania; catalina.gradinaru@tuiasi.ro; 2 Faculty of Civil Engineering, Transilvania University of Brașov, 500152 Brașov, Romania; radu.m@unitbv.ro; 3 Faculty of Architecture “G.M. Cantacuzino”, “Gheorghe Asachi” Technical University of Iași, 700050 Iași, Romania; bogdan-vasile.serbanoiu@academic.tuiasi.ro

**Keywords:** agro-waste, sustainable building materials, ecological concrete, natural fibres based composites

## Abstract

The effects of the fly ash and of the sunflower stalks and corn cobs within a cement-matrix composite were studied under the aspects of density, compressive strength, splitting tensile strength, elasticity modulus, and resistance to repeated freeze-thaw cycles. In the research were developed 20 recipes of cement-based composite, including the reference composite. Fly ash was used as partial cement replacement (10, 20 and 30% by volume), and the vegetal aggregates made by corn cobs and sunflower stalks as partial replacement of the mineral aggregates (25 and 50% by volume). The study results revealed that a lightweight composite can be obtained with 50% of vegetal aggregates, and the fly ash, no matter its percentage, enhanced the compressive strength and splitting tensile strength of the compositions with 50% of sunflower aggregates and the freeze-thaw resistance of all compositions with sunflower stalks.

## 1. Introduction

Environmental sustainability is an essential issue that should ensure the preservation of natural surroundings and the obtaining of economic benefits from decreasing the costs of construction materials and increasing energy efficiency. A sustainable building means that its emissions are reduced to a minimum or even to zero; to accomplish this goal, there must be different sustainable strategies used such as use of renewable raw materials or raw materials made by wastes and/or energy efficient building materials [1]. Nowadays, a very well-known fact is that concrete is the most popular building material. It has reached a consumption of 12 billion tons per year, and it is estimated to have a production rate of about 3–3.8 tons per inhabitant [2], which involves a proportionate cement production with short and long term consequences for human health and living beings in general [3,4], and significant emissions of CO_2_ and other greenhouse gases [5]. The cement production process involves a calcination reaction of calcium carbonate which generates around 0.55 tons of CO_2_, another 0.40 tons being emitted due to the fossil fuels combustion. Also, cement production is a powerful energy consuming process; this consumption being estimated as about 5% of global industrial energy consumption [6].

Using fly ash in concrete as a partial substitute of the cement in its composition is a solution for making a sustainable building material with a significant ecological character, a solution long researched and already applied in the world. Fly ash or solid fuel ash is a byproduct of pulverized coal combustion used in power plants. The fly ash particles are generally spherical and have sizes between 0.5–100 microns [7,8]; they are mostly composed of silicon dioxide [8]. The physical and chemical properties of fly ash can vary from plant to plant, mainly due to differences in the coal source [8,9]. In cementitious mixes, fly ash develops a minimal strength at initial ages [8,9]. The pozzolanic reaction depends on the fineness and the size distribution of its particles, and on the silica content also [9]. Fly ash influences the properties of fresh concrete, like the workability, water demand, setting time, segregation, bleeding [8,9,10]. The use of up 50% fly ash dosage in the binder mix increases the concrete workability [9]. Fly ash is recommended to be used in fiber-reinforced concrete to equilibrate the workability loss due to fibers use [9]. It extends the setting time of the concrete in curing temperatures up to 30 °C; the fly ash reactivity increases with the curing temperature increase up to 90 °C [9]. The increasing dosage of fly ash diminishes the concrete segregation and bleeding since it determines improved cohesiveness, smaller water content, and permeability due to its fine particles and low specific gravity [9,10]. The hydration heat decreases as the fly ash content in concrete mixes increases [7,9,10]. In hardened concrete, fly ash rates of up to 50% improve the splitting tensile strength and flexural strength [9]. Regarding the compressive strength, it decreases the initial-age strength [9,11], but in long periods, fly ash dosages up to 25–35% determine compressive strength and elasticity modulus improvements [9]. The concrete behavior to sulfate attack, thermal cracking, and alkali-silica expansion is improved with a dosage of 25–30% fly ash [9,10].

The aggregate extraction represents one of the most important mining industries in the world with a yearly production of around 16.5 billion tones valued at over $70 billion [12]. The environmental impact of aggregate mining activities is limited by direct regulations such as safety and health standards like the Federal Clean Water Act of Clear Air Amendments (in the United States of America), Environment Action Program (in the European Union), or by indirect regulations like the Fish a Wildlife Coordination Act, the Migratory Bird Treaty Act, The Endangered Species Act, Coastal Zone Management Act, National Environmental Policy Act, and so on. Commonly, there are regulations regarding air and water quality, non-coal surface mining, or zoning coats which impose restrictions or do not allow aggregate mining [12].

There are signals that building industry already started to follow sustainable principles in their design projects, using sustainable design concepts and materials [1], but there is still a lot of work to do. Plant based building materials have adequate properties which accomplish the modern buildings design requirements such as sustainability in construction and maintenance, efficiency in operating, and carbon footprint reduction. The vegetal raw material is an easily renewable one; it offers improved acoustic and thermal insulation and absorbs carbon dioxide from the environment. The high porosity of vegetal aggregates and their low apparent density recommend them to be used for producing a concrete with very good acoustic and hydro-thermal behavior compared to the conventional one with mineral aggregates (sand and gravel). The vegetal aggregates have a high flexibility which results in an increased ductility beyond the point when the maximum mechanical strength was reached, and a high deformability on the pressure [13]. As alternative materials, the vegetal aggregates bring benefits from health, ecologic, comfort, and sustainability perspectives. Also, global warming, life cycle issues, and energy savings represent factors which encourage the development of plant-based building materials. The requirements from the E.U. thermal codes specify that, starting from 2020, the energy consumption of all new buildings must decrease from 200 to 15 kWh/m^2^/yr, arguing for the development of plant-based materials due to their thermal insulation properties and positive effect on the environment [13].

Corn cob/sunflower cement-matrix composites represent environmentally preferable material due to the combination of waste content, low emissions, and locally derived materials. The sustainable development on the local level is in interdependence with worldwide problems like global warming or mineral resource exhaustion [13].

Over time, the main uses of corn cob were as animal feed (ground together with maize and other cereal grains) or as a firewood source for domestic heating and cooking, while the sunflower stalks represent agricultural waste that is put under the furrow plow.

Maize represents a plant with the most geographically ubiquitous crop. Its cultivated area was about 178 × 10^6^ ha in 2017, being the second most prevalent crop in the world in terms of area, after wheat, but the biggest in terms of production per ha (5.9 tons/ha), and global quantity (1042.4 × 10^6^ tons) [14]. Maize represents a major crop in China, USA, South Africa, and Eastern Europe. Sunflower has a smaller crop area than maize (26.03 × 10^6^ ha), with 2.07 tons/ha yield and a production value of around 53.94.41 × 10^6^ tons [15]. It represents a major crop especially in Southern South America, Southern Europe, European Russia, and Southern Africa [16].

Generally, vegetal dry matter (DM) consists of gross ash and organic matter. The organic matter consists of gross protein, gross fat, gross cellulose, and non-nitrogenous extract (NNE) or lignin. The main components of a plant cell are cellulose and lignin. According to the Table 1, it is observed that corn cobs contain an important rate of lignin, about 60% of the total organic matter, representing an almost double percentage that of the one found in the sunflower stalks. Regarding the cellulose quantity, the sunflower stalks are the ones with the highest percentage, 52.41% of the total organic substance, compared to 36.16% found in corn cobs. Given these values and the fact that cellulose has a stronger hydrophilic character than lignin, it can be concluded that sunflower stalks have a capacity for higher absorption of liquids than corn cobs. At the same time, given that lignin acts as a barrier in the plant structure against insects and fungi, corn cobs have the advantage of increased resistance to this type of attack [17].

Maize and sunflower are convenient in terms of money and processing technologies, local resources being used, without any high additional costs. Corn cobs and sunflower stalks are plant waste that are of interest to the building industry, being a widely available resource, inexpensive, annually renewable, with qualities in terms of thermal [18,19,20,21], and acoustic insulation [22,23]. Pinto et al., 2012 [19] found that the corn cobs have similarities as chemical elementary composition with expanded clay and as microstructure with expanded polystyrene and expanded clay. Nozahic et al., 2012 [24] found similarities between sunflower stalks and hemp regarding the morphology, density, chemical composition, and mechanical performance in the resulting material made by vegetal aggregates and a pumice-lime binder.

Compared to the traditional concrete, the one with lignocellulose matter is characterized by a higher permeability and lower density, thermal conductivity, and compressive strength. Considering the last issue, these types of concrete or cement based composites are not recommended as bearing building materials, but they are suitable as insulation ones, reducing the energy needs for heating and cooling the buildings [24]. They also have promising soundproofing characteristics [22].

As comparative literature, to our knowledge, very few papers analyzed the behavior of these natural materials into a cement based composite and none in the manner this study did, as regards the treatment of vegetal aggregates and their percentage in the composite composition. Życiński et al., 2020 [25] studied a concrete with 3 wt%, 5 wt%, and 10 wt% of simple ground corncob, without any treatment applied to the vegetal raw material. They obtained a decrease in density of around 7%, in compressive strength of around 50% and in splitting tensile strength of almost 24%, compared to reference. Pinto et al., 2012 [20] developed a lightweight concrete with corn cobs broken in large pieces with a mix ratio of 6:1:1 and 3:1:1 (aggregates: Portland cement: water). Their compositions registered a density of 382 kg/m^3^ and 778 kg/m^3^, respectively, and a compressive strength of 0.120 N/mm^2^ and 0.392 N/mm^2^, respectively. Pinto et al., 2012 [20] compared these recipes of corncob with similar ones made with expanded clay and they observed that corn cobs formed a material 34% lighter. They concluded that corn cob aggregates can provide sustainable and economic advantages as lightweight aggregates. Binici et al., 2016 [26] made a material from grounded corn cobs (named corn stalks in their research), cement, gypsum, NaOH, and aluminum dust with density between 0.5–0.8 kg/m^3^ and a compressive strength between 0.11–0.29 N/mm^2^. Nozahic et al., 2012 [24] developed a green material with grounded sunflower stalks and a pumice-lime binder instead of cement and used as reference a material made by the same method but with hemp. Their material with sunflower aggregates registered a density of 1084 kg/m^3^, smaller than the reference (1184 kg/m^3^) and a compressive strength of 2.52 N/mm^2^ also smaller than the reference (2.77 N/mm^2^). Mati-Baouche et al., 2014 [27] developed a material from sunflower stalks and chitosan and obtained a compressive strength of 2 N/mm^2^.

The aim of this study was to analyze the properties on some recipes of cement-matrix composites made with fly ash, corn cobs and sunflower stalks, in 19 different combinations. The bio-based composite variants implied the use of fly ash as partial cement replacement (10, 20 and 30% by volume) and of vegetal aggregates made by corn cobs/sunflower stalks as partial replacement (25 and 50% by volume) of the mineral aggregates. There were made recipes by applying only the partial cement replacement, by applying only the partial mineral aggregates replacement, and by applying both. The obtained bio-based composites were comparatively analyzed with a reference composite without any replacement and, also, between them. The analyzed characteristics were the density, compressive strength, splitting tensile strength, modulus of elasticity and the resistance to 50 cycles of freeze-thaw.

The topic of this research is important since it analyses the possibility to reduce the consumption of the natural mineral resources used in the most used material in building industry, the concrete, by using vegetal aggregates instead, which are renewable and widely spread throughout the world. Cement-matrix composites with vegetal aggregates are ecological alternatives that meet the sustainability requirements for a healthy environment, offering non-pollutant variants in the context of a highly industrialized society.

## 2. Materials and Methods

### 2.1. Materials

The cement-matrix composites were developed starting on a reference composition (RC) that was a conventional micro concrete of C25/30 strength class, made with sand and gravel aggregates with a maximum size of 8 mm, according to NE 012/1-2007 [28]. The RC composition involved the use of a Portland CEM II/A-LL42.5R cement (HeidelbergCement Romania, Bucharest, Romania), two types of river aggregates (sand up to 4 mm in diameter and gravel of 4–8 mm sort), as well as a superplasticizer and deflocculating additive based on polycarboxylateter, its ingredients being in accordance with SR EN 934-1:2008 [29] and which corresponds to SR EN 934-2:2012 [30] stipulations, and a rhodanide-based hardening accelerator additive, its ingredients being in accordance with SR EN 934-1:2008 [29] and SR EN 934-2:2012 [30] stipulations. The water/binder ratio used was 0.43. It served as a common starting point for developing cement-based composite blends by partial replacement of cement with fly ash (in 10, 20, and 30% by volume) and/or by partial replacement of mineral aggregates with two types of vegetal aggregates, namely sunflower stalks aggregates and corn cobs aggregates (in 25 and 50% by volume).

### 2.2. Methods

#### 2.2.1. Fly Ash and Cement Analysis

The fly ash and the cement were by Scanning Electron Microscopy coupled with Energy Dispersive X-Ray (Gatan’s OnPoint™ detector, Gatan Inc., Warrendale, PA, USA), and an EDAX Genesis^TM^ APEX 2 EDX System (AMETEK Inc., Paoli, PA, USA).

#### 2.2.2. Vegetal Aggregates Preparation

**Figure 1:**
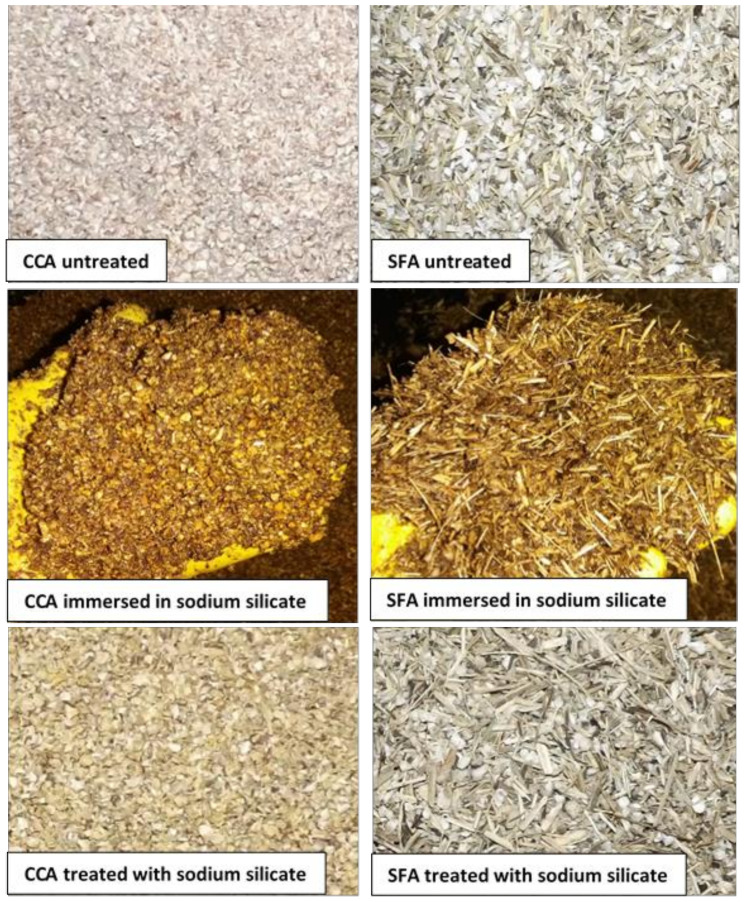
The corn cobs aggregates (CCA) and sunflower aggregates (SFA) preparation.

The corn cobs and sunflower stalks, respectively, were grounded with a mill for animal feed crushing; the corn cobs resulted in granules with 2–6 mm diameter, and the sunflower stalks in granules with same dimensions and fibers up to 25 mm in length (Figure 1). This crushed raw material was treated with a solution of 40% sodium silicate (glass water) by immersion, in order to reduce their absorption capacity and improve rot resistance, as well as to obtain a better interface compatibility with the cement matrix; then, the vegetal aggregates were dried on a heated plane surface with temperature of 52 ± 2 °C till constant mass. The dried vegetal aggregates were used in the composite mixes. The method for obtaining vegetal aggregates is presented in detail in previous research, Grădinaru et al., 2020 [31].

#### 2.2.3. Composite Mixes Preparation

To conduct this study, cement-based composite mixes with 0, 10, 20, and 30% fly ash were developed as a cement substitute. These served as references for the subsequent realization of other cement-based composites which involved maintaining the same matrix, but replacing the mineral (river) aggregates with vegetal ones in proportions of 25 and 50% by volume. Table 2 shows the particular structure of the developed mixes.

**Figure 2:**
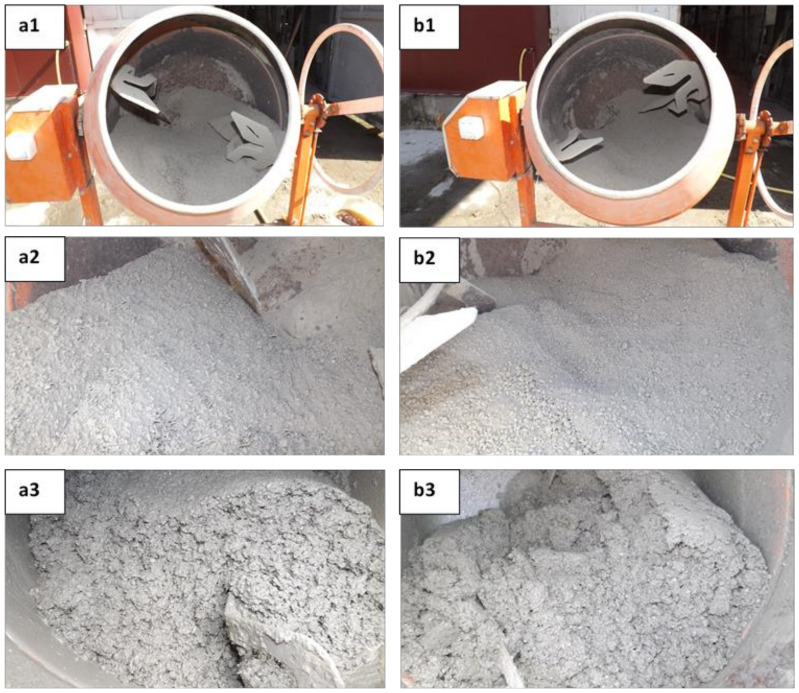
Composition preparation: (**a1**) dry components of sunflower stalks composites, mixed within portable electric concrete mixer; (**a2**) dry components of sunflower stalks composites, close up view; (**a3**) sunflower stalks composite in fresh state; (**b1**) dry components of corn cobs composites, mixed within portable electric concrete mixer; (**b2**) dry components of corn cobs composites, close up view; (**b3**) corn cobs composite in fresh state.

For composite mixes preparation (Figure 2), a portable electric concrete mixer was used. First the dry components were mixed (mineral aggregates, vegetal aggregates, cementitious binder), and then the water mixed with the additives was added, continuing blending until a homogeneous composition was obtained.

**Figure 3:**
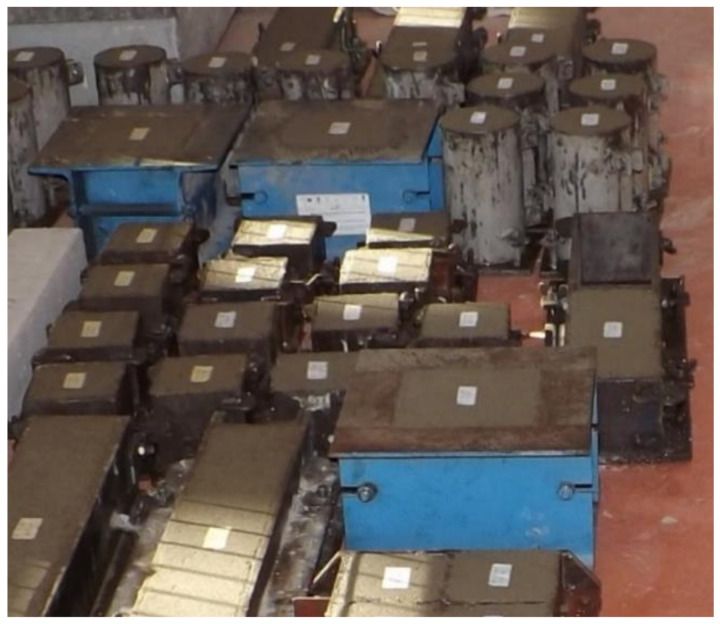
Composite specimens of a developed mix, in molds.

The developed composites were cast in 150 mm cube molds for the compression test, in 100 mm cube molds for repeated freeze-thaw cycles testing and in cylinder molds with 100 mm diameter and 200 mm length for splitting tensile testing and for measuring the modulus of elasticity (Figure 3). The concrete compositions were made according to NE 012/1-2007 [28]. Concrete specimens were unmolded after 24 h, then they were cured for 28 days in ambient conditions, with air temperature of 20 ± 5 °C and relative humidity of 45 ± 10%.

#### 2.2.4. Composite Specimens Properties

##### Density Determination of the Fresh and Hardened Cement-Based Composite

The determination of the bulk density consists in determining the mass of a fresh/hardened composite sample and its ratio to the sample volume in the compacted state, in accordance with EN 12350-6:2019 [32] and EN 12390-7:2019 [33], respectively. To determine this parameter, cubic metal containers with sides of 150 mm were used. Three samples were tested, and the average result was calculated to assess the density for each concrete mix. The values graph from the results section contains the error bars accordingly assessed.

##### Compressive Strength

**Figure 4:**
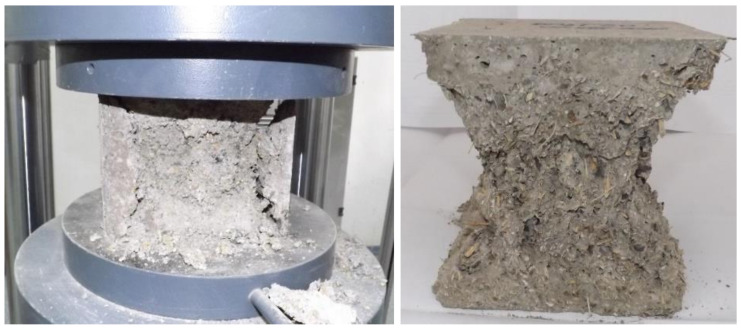
Compressive strength testing.

The cement-based composite specimens were tested at mono-axial compression according to EN 12390-3:2019 [34], at 28 days (Figure 4). The specimens were cubes with sides of 150 mm. Three samples were tested, and the average result was calculated to assess the compressive strength for each concrete mix. The values graph from the results section contains the error bars assessed accordingly.

##### Splitting Tensile Strength

**Figure 5:**
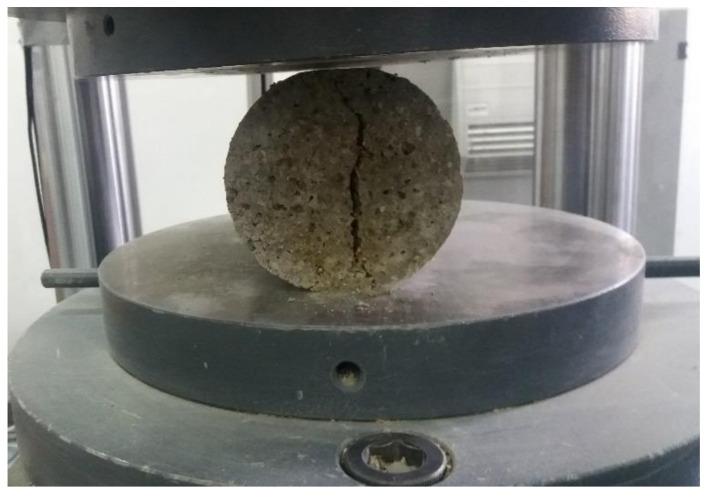
Splitting tensile testing.

The splitting tensile strength test consisted of compressing cylinders of 200 mm length and 100 mm in diameter after two diametrically opposed generators, according to standard EN 12390-6: 2009 [35] (Figure 5). Three samples were tested, and the average result was calculated to assess the splitting tensile strength for each concrete mix. The values graph from the results section contains the error bars accordingly assessed.

##### Static Modulus of Elasticity Determination of the Cement-Based Composite

For elasticity modulus determination, the second method from EN 13412: 2006 standard [36] was applied, because it is intended for products based on cement binders.

**Figure 6:**
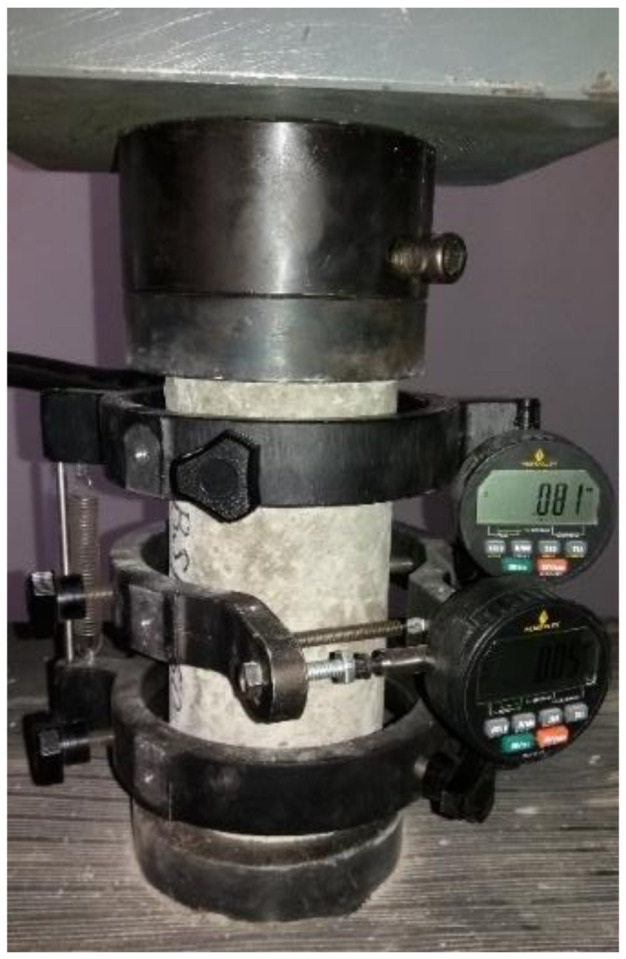
Modulus of elasticity testing.

Modulus of elasticity is determined by strain change measuring (Figure 6) in the composite sample when loaded, to produce a stress between 0.5 N/mm^2^ and 1/3 of the compressive strength value of the sample determined previously according to EN 12390-3:2019 [34]. Three samples were tested, and the average result was calculated to assess the modulus of elasticity for each concrete mix. The values graph from the results section contains the error bars accordingly assessed.

##### Determination of the Resistance of the Composites to Repeated Freeze-Thaw Cycles

The determination of the freeze-thaw resistance was performed according to SR 3518: 2009 [37]. This standard is used when is analyzed the performance of concretes after they have been subjected to repeated freeze-thaw cycles. The standard assumes the existence of a destructive method for determining these performances by compressive strength testing, and a non-destructive method, which involves measuring the relative dynamic modulus of elasticity. As the non-destructive method is recommended to be applied in the case of normal density concrete, the destructive method was used in the research carried out in this paper, as it is recommended in the case of both normal density and lightweight concrete. This method involved the realization of cube-type specimens with 100 mm sides, the test being performed after the curing period of 28 days. The developed composites were subjected to 50 freeze-thaw cycles. For this number of cycles, according to the specification from the mentioned standard, the production of six specimens from each composition was taken into account, three of them being control specimens and the other three were effectively subjected to the freeze-thaw cycles. The values graph from the results section contains the error bars accordingly assessed.

The procedure required that all samples be placed in a water bath of 20 ± 5 °C temperature, the water level being initially up to ¼ from the height of the specimens. After 24 h, the water level is raised up to ½ from the height of the specimens, then after another 24 h up to ¾, and then after the same time the specimens are kept at least 20 mm below the water level. To achieve complete saturation, they are kept completely immersed in water for 24 h.

**Figure 7:**
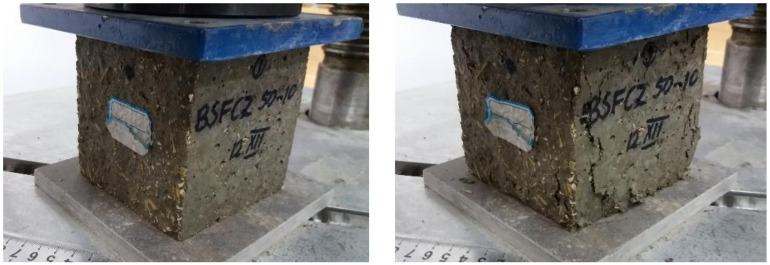
Composite specimen subjected to freeze-thaw cycles, before and after compressive strength testing.

**Figure 8:**
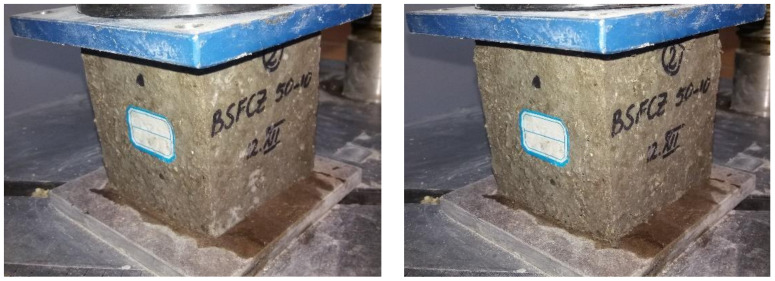
Composite control specimen, before and after compressive strength testing.

The three control specimens remain in the water bath all the time while the other three are placed alternatively in a cold room that maintains the temperature at −17 ± 2 °C for minimum 30 min at the end of the freeze stage, and then in a water bath. A cycle consists in 4 h of freezing, and 4 h of thawing in a water bath at a temperature of 20 ± 5 °C. At the final of 50 cycles, all the specimens are tested for compression, including the control specimens that were kept always in water (Figure 7 and Figure 8). The loss of compressive strength is calculated and analyzed.

## 3. Results and Discussions

### 3.1. Fly Ash and Cement

#### 3.1.1. Cement

The cement used in this research was cement type II, its full name being CEM II/A-LL 42.5R (notations according to EN 197-1: 2011 [38]).

**Figure 9:**
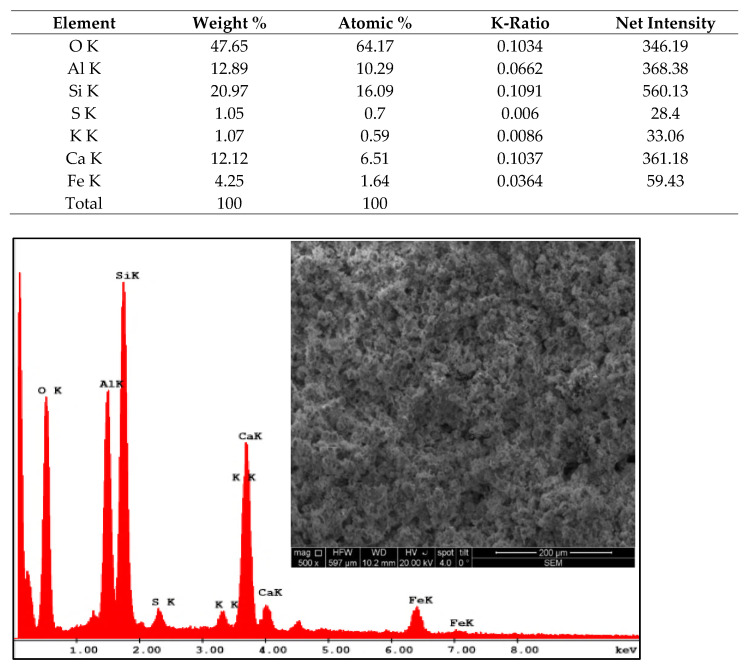
Elementary chemical analysis of CEM II/A-LL cement and SEM image of particles at a magnification rate of 500×.

The EDX analysis (Figure 9) revealed the following elementary chemical composition, related to the total mass of the cement used in the research: Oxygen (O)-47.65%, Aluminum (Al)—12.89%, Silicon (Si)—20.97%, Sulfur (S)—1.05%, Potassium (K)—1.07%, Calcium (Ca)—12.12% Iron (Fe)—4.25%.

The elementary chemical composition of CEM II / A-LL 42.5R cement, expressed as developed area, is as follows: Oxygen (O)—64.17%, Aluminum (Al)—10.29%, Silicon (Si)—16.09%, Sulfur (S)—0.70%, Potassium (K)—0.59%, Calcium (Ca)—6.51%, Iron (Fe)—1.64%.

#### 3.1.2. Fly Ash

The fly ash from the thermal power plant used in this research was the one resulting from the CET Holboca Iasi plant, Romania, and was used as a substitute for 10, 20, and 30% of the cement volume.

**Figure 10:**
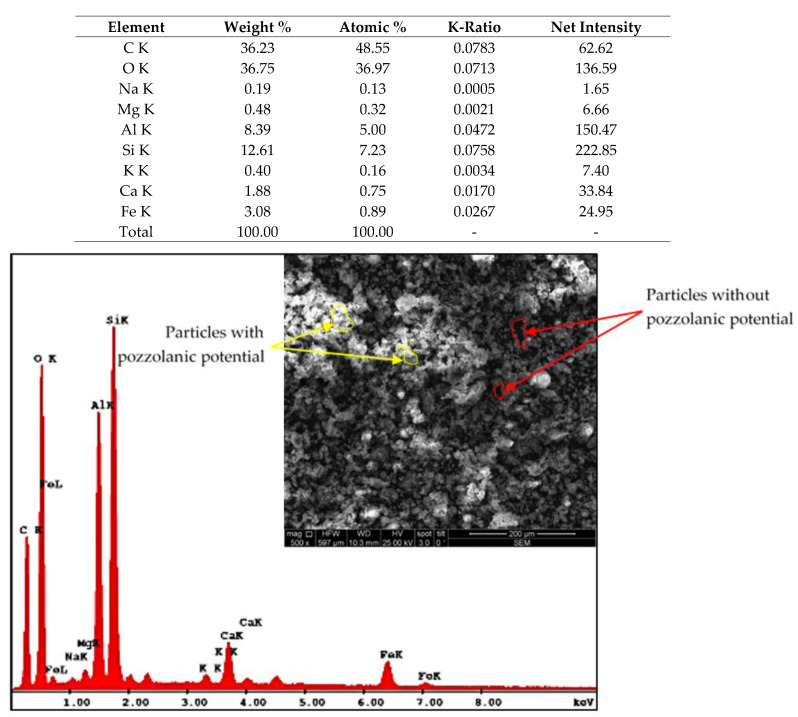
Elementary chemical analysis of fly ash produced by CET Holboca Iași and SEM image of particles at a magnification rate of 500×.

The EDX analysis (Figure 10) revealed the following elementary chemical composition, expressed in mass percentages, of the fly ash used in the realization of the concrete recipes developed in this study: Carbon (C)—36.23%, Oxygen (O)—36.75%, Sodium (Na)—0.19%, Magnesium (Mg)—0.48%, Aluminum (Al)—8.39%, Silicon (Si)—12.61%, Potassium (K)—0.40 %, Calcium (Ca)—1.88%, Iron (Fe)—3.08%.

The elementary chemical composition of fly ash, expressed as unfolded area, is as follows: Carbon (C)—48.55%, Oxygen (O)—36.97%, Sodium (Na)—0.13%, Magnesium (Mg)—0.32%, Aluminum (Al)—5.00%, Silicon (Si)—7.23%, Potassium (K)—0.16%, Calcium (Ca)—0.75%, Iron (Fe)—0.89%.

In Figure 10 the micrograph of the fly ash produced by CET Holboca Iași can be observed, according to the SEM method at a magnification level of 500×. Analyzing comparatively with the cement used in the research (Figure 9), it is found that the fly ash has a structure with larger and rarer particles. It is also easy to see the relatively significant share of particles with pozzolanic potential, gray in color.

As far as chemical composition major differences, it can be mentioned that the fly ash contains C at a quite high rate (36.23%), and smaller rates of O (with almost 11%), Ca (with around 10%), Si (with around 8%), Al (with 4.5%), and Fe (with around 1%) than the cement.

It is a very known fact that fly ash properties differs from one power plant to another, depending on the burned fuel characteristics, but it always contains unburned carbon. The carbon content has a negative influence on the mechanical properties of the concrete. The main elements that give higher pozzolanic qualities to fly ash are alumina, silica, and iron [9].

### 3.2. Composite Specimens Properties

#### 3.2.1. Density

**Figure 11:**
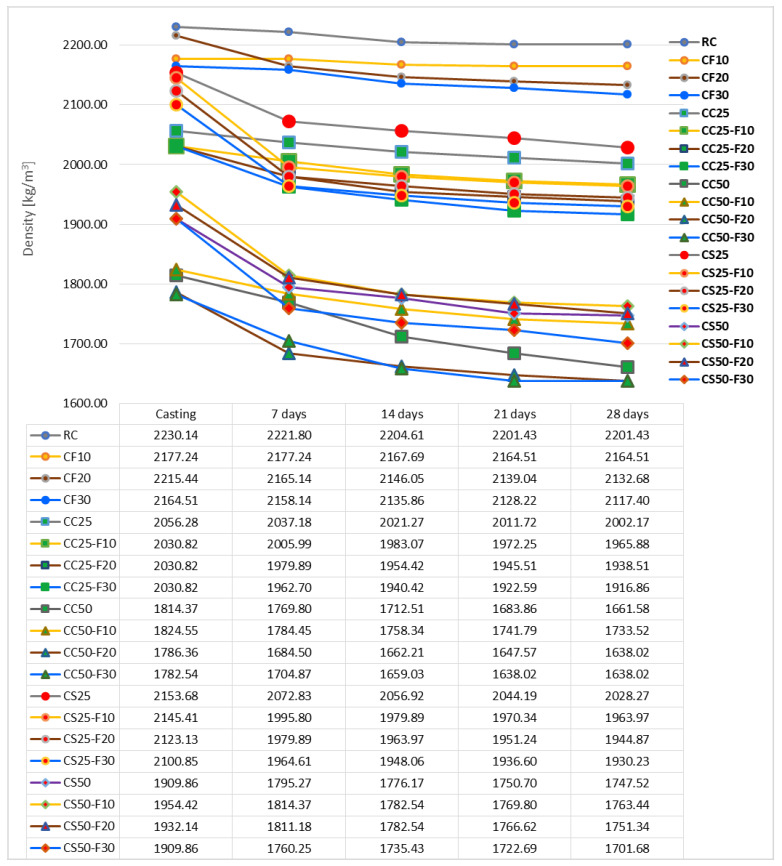
Density evolution of the composites with fly ash, corn cobs, sunflower stalks during the 28 days of curing [kg/m^3^].

The evolution of the specimen density during the curing period of 28 days is represented in Figure 11.

From its analysis, it can be seen that the curve inclination is different for each composite variant. The smoothest slope was recorded by CF10, which means that this recipe had the lowest water loss by evaporation. Given that the water/binder ratio was the same, CF20 recorded the steepest decrease in its density, especially in the first seven days after casting.

During the 28 days of curing, RC registered a density decrease by 1.2% and CC25 by 2.63%. The smallest decrease in density was recorded by CF10, with 0.58% and the largest decrease, by CC25-F30, with 5.6%. From the group of CF composites, the largest decrease was achieved by CF20, 3.74%. From the CC-F group, the smallest decrease was registered by CC25-F10, 3.2%. In conclusion, the fly ash caused the density of the composite with corn cobs aggregates to decrease.

Regarding the CS composites, during the curing period of 28 days, these registered a density decrease of approx. 6 and 8.5%, in case of the replacement of mineral aggregates by 25 and 50%, respectively. In the case of CS25, the partial replacement of cement with fly ash in proportions of 10, 20, and 30%, resulted in a greater decrease in density during this period, about 8–8.5%. In the case of CS50, the replacement of cement with fly ash in the same proportions as in the case of CS25 also determined a greater decrease in density by approx. 9.4–11%. Thus, it can be concluded that fly ash also contributed to the decrease in density of CS composites.

**Figure 12:**
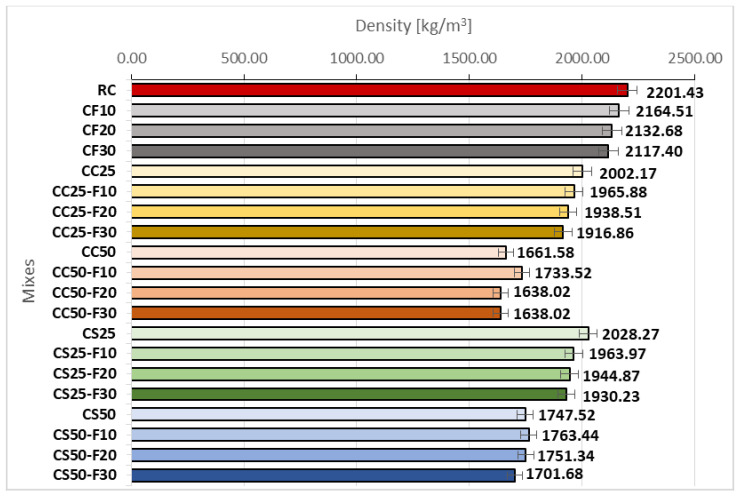
Density of composites with fly ash, corn cobs, and sunflower stalks, measured 28 days after casting [kg/m^3^].

Figure 12 shows the density of composites with corn cob aggregates, sunflower stalks and fly ash at 28 days.

According to Figure 12, the volume increase of fly ash to the detriment of the cement, led to a decrease in the composites’ density. The lowest density in the CF group was recorded by CF30, with a value of 2117.40 kg/m^3^, 3.83% lower than that of RC. CC25 recorded a decrease in density by around 9% compared to RC. It can be seen that the association of fly ash with corn cob aggregates had significant positive effects, as the CC-F group recorded the lowest density of all, below 2000 kg/m^3^, with a decrease between 13 and 11% compared to RC. CC50-F10 registered the smallest decrease in density, with 4.9% (Figure 12). The CC50-F group recorded density values lower than 1770 kg/m^3^, the decrease being between 25.59 and 21.25%, compared to RC.

CS25 recorded a density about 8% lower than RC. From the Figure 12 analysis it is observed that the partial replacement of cement with fly ash (10, 20, 30%), in parallel with the replacement of conventional aggregates with 25% sunflower aggregates caused the density decrease by approx. 3–4.5%, compared to the value recorded by CS25.

Replacing 50% of sand and gravel aggregates with sunflower aggregates reduced the RC density by approx. 20%. Further, the replacement of 10, 20, or 30% of the volume of cement with fly ash did not cause significant variations in the density of CS50.

**Figure 13:**
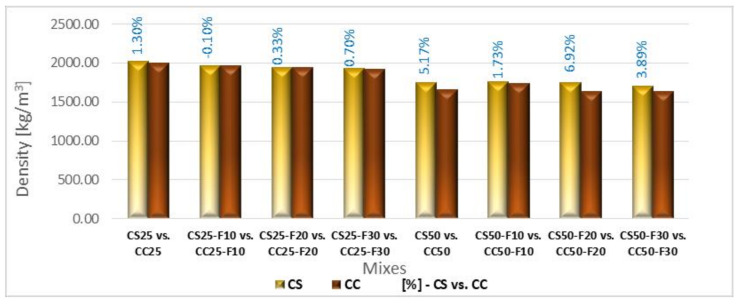
Density variation of composites with aggregates from sunflower stalks versus composites with corn cob aggregates [%].

Analyzing comparatively CS to CC (Figure 13), it is observed approximately similar densities in the case of 25% replacing the classical stone aggregates with vegetal ones. The same observation can be made in the case of cement replacement with fly ash, for the same rate of replacement of aggregates. In the case of 50% replacement rate, sunflower composites had higher densities than those with corn cobs. Thus, the use of 10% fly ash and 50% plant aggregates led to a 1.73% higher density of the composites with sunflower stems, compared to those with corn cobs. Increasing the fly ash percentage to 30% led to a bigger density difference between the two types of plant composites of about 4%, and the use of a percentage of 20% fly ash, to a difference of about 7%. The density difference between the two variants of plant composites with 50% rate of replacement of aggregates, without fly ash, was approx. 5%, the lightest being the one with corn cobs. In conclusion, the composite with 50% aggregates of corn cobs and fly ash obtained the lowest densities, between 1638 kg/m^3^ and 1733 kg/m^3^. The densities recorded by the composite with 50% sunflower stalks, with or without fly ash, did not fall below 1700 kg/m^3^. In the case of replacing the aggregates in a percentage of 25%, densities between approx. 1916 and 2028 kg/m^3^ were obtained.

The 28 days density of CC, respectively CS, decreased along the plant material rate in the composition increased. Lightweight concrete can be obtained when are used rates over 50% of plant matter.

The CS compositions recorded bigger densities than CC ones. The organic chemical composition of plant aggregates is responsible for these results. Even the sunflower stalks aggregates registered a smaller bulk density than the corn cobs ones, they have higher cellulose content that determines a bigger absorption of cement paste in their structure than in the case of corn cobs; in this way, a more compact packaging is obtained and a bigger density of the vegetal composite. Corn cobs have a significant content of lignin that is responsible for a smaller absorption capacity; this fact results in a lighter cement-based composite. In conclusion, the internal structure of the vegetal aggregates plays an important role in the density level of the cement-based composites.

According to C155-2013 [39], lightweight concrete can be classified in seven classes, from D0.8, up to D2.0. In Table 3 is presented the classification obtained by the cement-based composite compositions developed and analyzed.

#### 3.2.2. Compressive Strength

**Figure 14:**
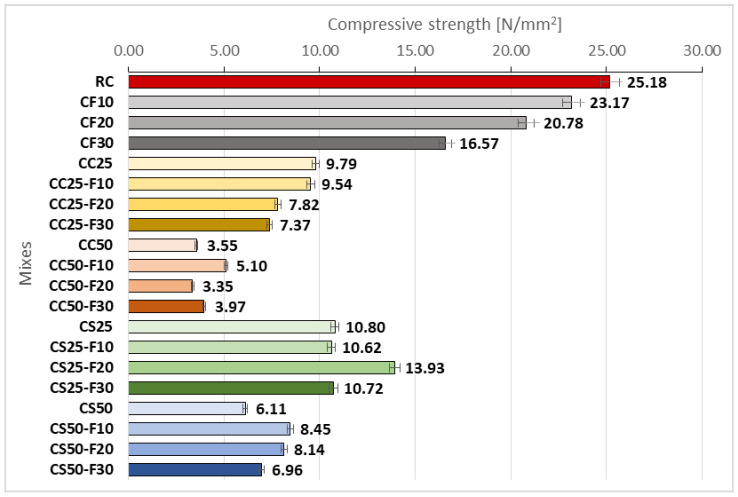
Compressive strength of the cement-based composites [N/mm^2^].

The compressive strength of the analyzed compositions is shown in Figure 14. An evident observation is that it decreased as the fly ash rate increased. For a replacement percentage of 10% of the cement volume with fly ash, the material obtained had a compressive strength 8% lower than RC. For a replacement percentage of 20%, the decrease was 10%, while a replacement percentage of 30% led to a decrease in compressive strength by about 17%. Therefore, although the cement amount replaced increased in equal rates, the effect on the compressive strength of the composite was not at the same rates. However, the decrease in compressive strength of the composite as the amount of fly ash increases is due to the amount of carbon in the fly ash composition.

Replacing 25% of mineral aggregates volume with corn cob aggregates resulted in a sharp decrease in the compressive strength of the cement-based composite by 61.12% (Figure 14). The association of fly ash with corn cob aggregates led to a lower compressive strength than CC25, with 2.55–24.72%. Replacing 50% of mineral aggregates with corn cob aggregates resulted in an even greater decrease in compressive strength by up to 85.90%. The combination of fly ash with 50% corn cob aggregates led to an improvement in compressive strength when using a replacement rate of 10% and 30% of the cement volume, by 43.66% and 11.83%, respectively, compared to CC50. Regarding the compositions with sunflower stalks, there was a registered decrease of their compressive strength by about 57% in the case of 25% replacement of mineral aggregates, and by about 75% in the case of 50% replacement. Fly ash had the effect of improving this parameter by about 40% in the case of CS50-F10, by about 30% in the case of CS50-F10, and by about 14% in the case of CS50-F30.

**Figure 15:**
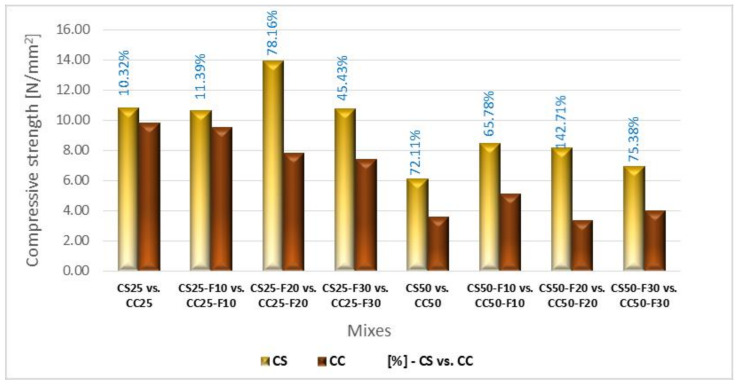
Compressive strength variation at the 28 days age of composites with sunflower stalks aggregates versus composites with corn cob aggregates [%].

Analyzing comparatively the composites with the two types of vegetal aggregates, from Figure 15 it can be observed that CS compositions registered bigger compressive strengths than CC ones. The replacement of 20% of the cement volume with fly ash had the best result, both in the case of replacing 25 and 50% of sand and gravel aggregates with vegetal ones. Thus, CS25-F20 obtained a higher compressive strength by approx. 78% compared to CC25-F20, and CS50-F20 143% higher than CC50-F20. Replacing 30% of the cement volume with fly ash resulted in a higher compressive strength of CS25-F30 by approx. 45% than CC25-F30. In the other cases of cement replacement with fly ash, the influence of the latter was insignificant on this parameter. Analyzing comparatively, the use of a volume of 20% fly ash, in the case of 25% replacement rate of the mineral aggregates, determined a difference between the compressive strength of concrete with sunflower and that with corn cobs of about eight-fold (78.16 vs. 10.32%, respectively). The same percentage of fly ash, when a replacement rate of 50% of mineral aggregates was applied, doubled the difference between the compressive strength of concrete with sunflower and that of corn cobs (142.71 vs. 72.11%).

According to NE 012/1-2007 [28], the strength classes of lightweight aggregate concretes are different from those of normal mass concrete. Table 4 shows the strength classes of lightweight aggregate concretes developed in this research. The notation of these classes consists of code LC (light concrete) followed by the minimum characteristic strength on cylinders / minimum strength on cubes, expressed in [N/mm^2^].

Among the compositions developed, CS25-F20 falls into the strength class LC12/13, and the concretes marked with CC25, CS25, CC25-F10, CS25-F10, CS25-F30, CS50-F10, CS50-F20 in the lower strength class, LC8/9. The rest of the composite compositions recorded compressive strengths below the level of LC8/9 strength class.

Due to the low density and, implicitly, the classification of these types of concrete in the category of lightweight ones, a series of practical applications becomes possible. Replacing traditional concrete with the one developed in this research may be an option where increased efficiency is sought in terms of other parameters than strength. The entire mass of a construction can be reduced by using these concretes in the form of leveling screeds, closing, and partitioning elements or thermal and sound insulation. Also, the low weight of some prefabricated concrete elements with vegetable aggregates makes them efficient in terms of transport and handling. Such prefabricated elements can be those for the infrastructure of communication routes and irrigation systems. 

#### 3.2.3. Splitting Tensile Strength

**Figure 16:**
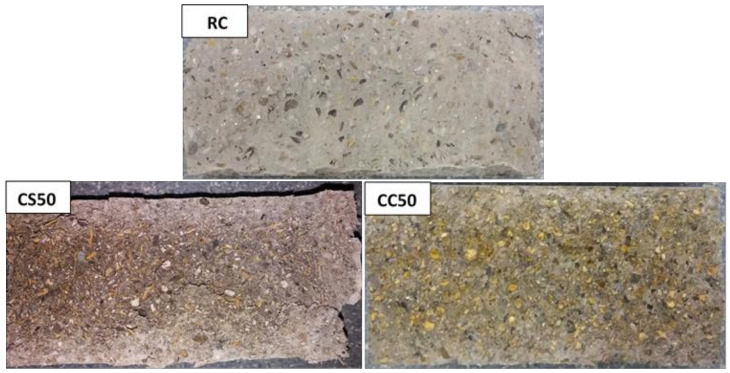
The RC, CS50, and CC50 cylinder’s section, after splitting tensile testing.

**Figure 17:**
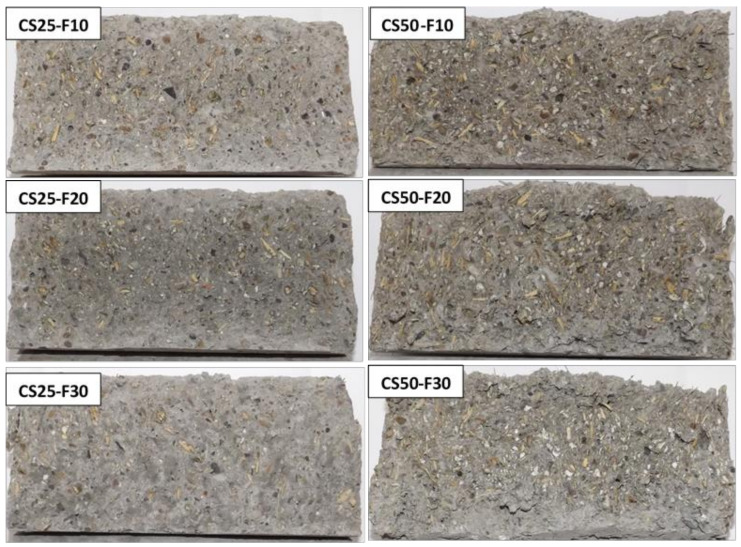
The cylinder’s section of CS-F group of composites, after splitting tensile testing.

**Figure 18:**
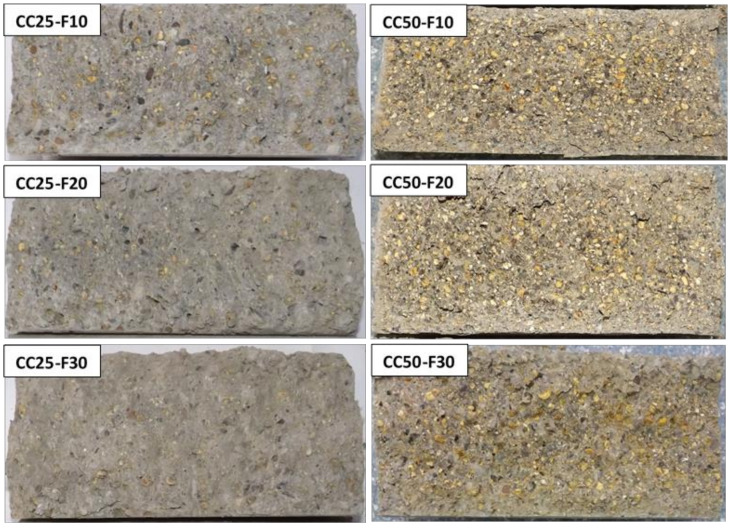
The cylinder’s section of CC-F group of composites, after splitting tensile testing.

In Figure 16, Figure 17 and Figure 18 and specimens in section are presented, as resulted after splitting tensile strength test. 

**Figure 19:**
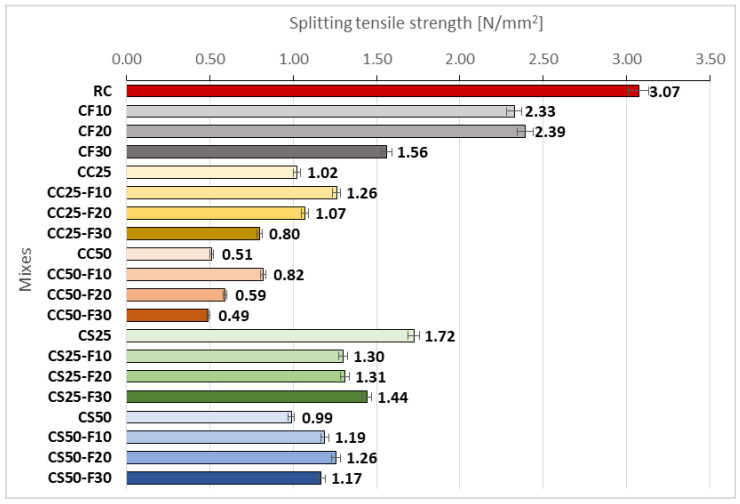
Splitting tensile strength of the composite samples [N/mm^2^].

The splitting tensile strength of the analyzed compositions is shown in Figure 19. Fly ash in a rate of 10% decreased the splitting tensile strength of the RC by around 24%. In the case of CF20, an improvement in splitting tensile strength of up to 2.6% has been observed, than the previous variant with 10% fly ash. A surplus of another 10% fly ash (CF30) led to a decrease of this parameter by approximately 35 and 50% compared to CF20 and RC, respectively.

By using fly ash in the CC25 composite, an improvement in splitting tensile strength was obtained, by 4.9 and 23.53%, when using 20 and 10% fly ash instead of cement, respectively (Figure 19).

The combination of fly ash with CC50 determined an improvement of the splitting tensile strength by 62.19 and by 15.68% in case of 10 and 20% replacement of the cement volume, respectively (Figure 19). The 10% replacement of the cement volume with fly ash had a positive influence on this parameter in the case of CC25, causing an increase of about 23%. The same positive influence was in the case of 20% replacing of the cement volume also, but the improvement obtained was smaller, of 4.90%. The use of a 30% replacement rate of fly ash with cement, on another hand, reduced the splitting tensile strength by more than 20% in CC25. In the case of CC50, fly ash improved their splitting tensile strength by about 60%, when was used a volume of 10% of the cement, and an increase of approx. 16% when was used a volume of 20% fly ash. The use of a fly ash-cement replacement rate of 30% resulted in a decrease of splitting tensile strength in the case of CC50, but to a lesser extent than in the case of CC25, by about 4%.

In the case of composites with 25% sunflower aggregates, the partial replacement of cement with fly ash caused the decrease of splitting tensile strength, but it led to increasing values as the fly ash rate was higher (Figure 19).

In the recipes with 50% sunflower aggregates, the fly ash improved the splitting tensile strength of all three variants of cement substitution. Thus, the application of a substitution rate of 10% of the cement led to an improved strength of about 38%, applying a substitution rate of 20% to an improvement of about 33%, and one of 30% to an improvement of about 14% (Figure 19).

**Figure 20:**
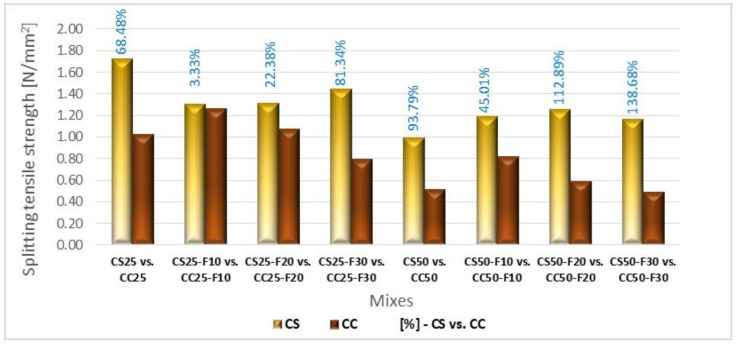
Splitting tensile strength variation of the composites with sunflower aggregates versus composites with corn cob aggregates [%].

Analyzing comparatively, the CS-F group registered higher splitting tensile strengths than those of CC-F group (Figure 20). In the case of the compositions with 25% vegetal matter, the sunflower aggregates led to a splitting tensile strength 68.50% higher than those with corn cobs aggregates. The use of 10 and 20% fly ash as a cement substitute reduced this difference to approx. 3 and 22%, respectively, also in favor of sunflower compositions. When a cement substitution rate of 30% was applied, there was an increase in this difference to approx. 81%.

In the case of 50% rate, the sunflower aggregates determined a higher splitting tensile strength by about 94% compared to corn cob aggregates. In the case of substitution of 10% of the cement volume with fly ash, this difference decreased to the level of 45%; however, in the case of substitution rates of 20 and 30%, this difference increased to about 113 and 139%, respectively. 

#### 3.2.4. Modulus of Elasticity

**Figure 21:**
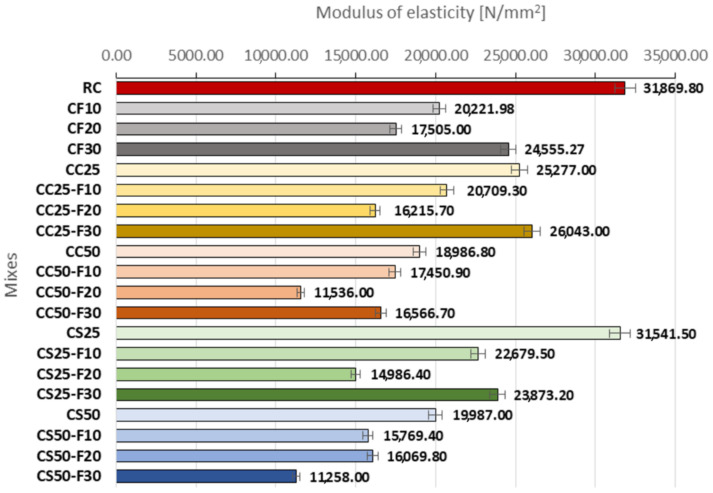
The modulus of elasticity of composite samples.

In Figure 21 are presented the modulus of elasticity values recorded by the composites made with aggregates of corn cobs, sunflower stalks, and fly ash. The compositions made with 10, 20, and 30% fly ash registered decreased values of this parameter. By replacing 30% of the cement volume with fly ash, the modulus of elasticity was lower than that of RC but higher than in the case of CF10 and CF20. The combination of these replacement rates of cement by fly ash with the replacement of mineral aggregates in proportions of 25 and 50% with corn cobs aggregates led to the same type of evolution of this parameter as in the case of the fly ash series, CF10, CF20, CF30. In the case of CC25-F30, the high percentage of fly ash determined a higher modulus of elasticity compared to both CC25 and CF30. A 50% replacement rate of mineral aggregates with corn cob aggregates resulted in the lowest values of the series of corn cobs composites.

The association of fly ash with CS25 determined the same type of evolution of the elasticity modulus as in the case of the fly ash standard series, CF10, CF20, CF30 (Figure 21). In the case of CS50, the 30% replacement rate of the cement volume with fly ash contributed to the decrease of the modulus of elasticity compared to the replacement rate of 20%; CS50-F30 registered the lowest value of this parameter from this series of composites with sunflower aggregates.

**Figure 22:**
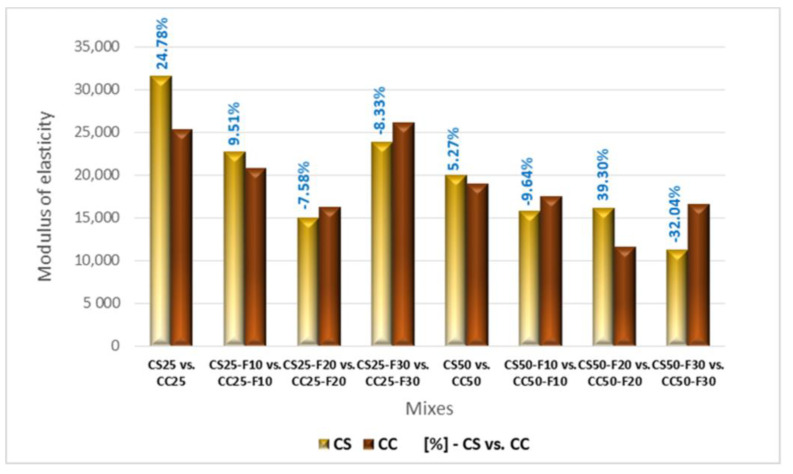
Variation of modulus of elasticity for composites with sunflower aggregates versus composites with corn cobs aggregates [%].

Analyzing comparatively the compositions with sunflower stalks and those with corn cobs (Figure 22), in the case of CC50-F20 and CS50-F20, CC25 and CS25 were registered the biggest differences between the values of the modulus of elasticity, in favor of the one with sunflower stalks. A significant difference between the two variants of vegetal aggregates was also recorded in the case of the recipe with 50% vegetal matter and 30% fly ash, but this time in favor of the composite with corn cobs.

#### 3.2.5. Resistance of Composites to Repeated Freeze-Thaw Cycles

**Figure 23:**
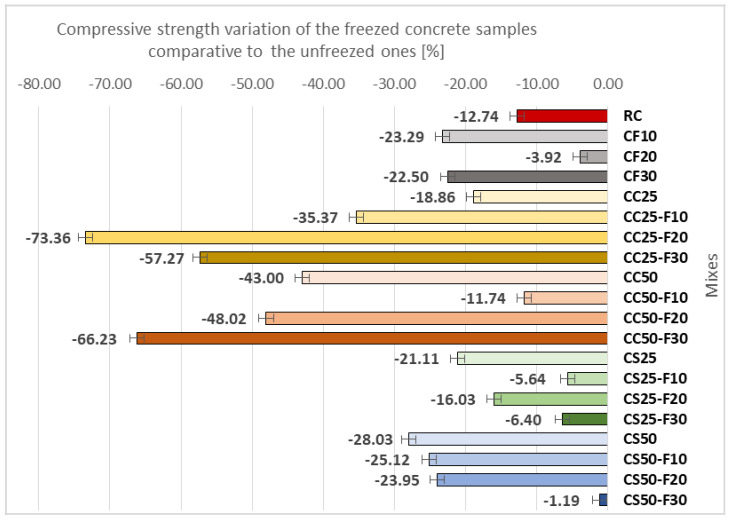
Variation in compressive strength of samples after testing at 50 freeze-thaw cycles, comparative to unfreezed samples [%].

Figure 23 shows the variation of the compressive strength of composite samples made with aggregates of corn cobs, sunflower stalks, and fly ash, subjected to 50 freeze-thaw cycles.

The replacement rate of 10 and 30% of the cement volume with fly ash caused a loss of compressive strength of the composites of about 23% (Figure 14). The 20% replacement of the cement volume with fly ash caused a much smaller decrease in compressive strength after freeze-thaw cycles of about 4%.

The combination of vegetal aggregates with fly ash led to a cumulative effect in terms of compressive strength loss of composites after 50 freeze-thaw cycles, in CC25. In the case of CC25-F10, the compressive strength diminished by about 35%, in the case of CC25-F30 by about 57%, and in the case of CC25-F20 by about 73%. In the case of the recipe with only 50% aggregates of corn cobs, the compressive strength decreased by 43%. The replacement of 10% of the cement volume with fly ash in the composition of CC50 resulted in a much lower loss of compressive strength, 11.74%. In the case of replacing 20% and 30% of the cement volume with fly ash, the loss of compressive strength increased to 48% in the case of CC50-F20, and to about 66% in the case of CC50-F30.

Sunflower composites registered a compressive strength decrease after 50 freeze-thaw cycles, of about 21% (Figure 23). The combination of CS25 and replacement of 10%, 20, and 30% of the cement volume with fly ash had positive effects on the loss of compressive strength following the freeze-thaw process. Thus, in the case of CS25-F20, the loss was 16%, in the case of CS25-F10, 5.64%, and in the case of CS25-F30, 6.4%. In CS50, the compressive strength decreased by 28%. The use of cement substitution rates of 10 and 20% led to a slight reduction of this decrease of compressive strength to 25.12 and 23.95%, respectively. The use of a cement substitution rate of 30% resulted in a substantial reduction in the loss of compressive strength following the 50 freeze-thaw cycles, CS50-F30 recording a lower compressive strength by 1.19% than the control sample.

In conclusion, CS50-F30 registered the most favorable results in terms of compressive strength after freeze-thaw testing of compositions with sunflower stalks (Figure 23).

**Figure 24:**
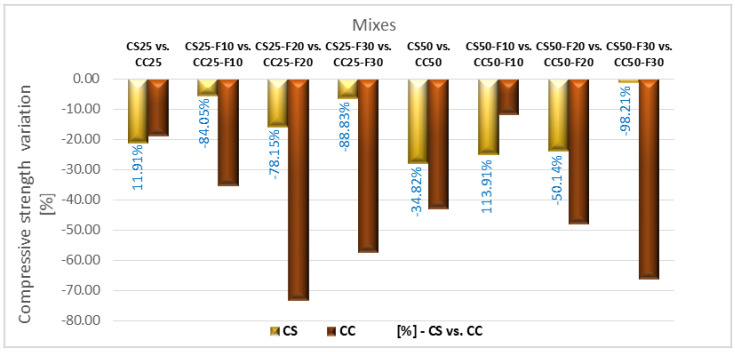
Variation of compressive strength loss of composites with sunflower aggregates versus composites with corn cobs aggregates, after 50 freeze-thaw cycles [%].

Comparing the sunflower and corn cob composites (Figure 24), the ones with sunflower stalks aggregates recorded, in general, a lower compressive strength loss than those with corn cobs aggregates, after the 50 freeze-thaw cycles. The parallel replacement of mineral aggregates, in proportion of 25% with vegetable aggregates with the cement replacement in proportion of 10, 20, and 30%, with fly ash, determined the smaller losses of compressive strength after the freeze–thaw process in the case of the composites with sunflower aggregates tested to freeze-thaw, these obtaining higher values of this parameter by around 84, 78, and 89%, respectively, compared to the compositions with corn cobs aggregates. The largest differences were recorded between the recipes with 50% vegetal aggregates in combination with 10 and 30% fly ash; in the case of the recipes with 10% fly ash, this was due to the more than double value of compressive strength loss of the sunflower composition compared to that of corn cob composition; in the case of 30% fly ash this was due to the insignificant value of the compressive strength loss recorded by the sunflower composition compared to the relatively high value recorded by the one with corn cobs.

## 4. Conclusions

The study involved the analysis of density, compressive strength, splitting tensile strength, elastic modulus, and resistance to repeated freeze-thaw cycles of a series of cement-based composites with fly ash as partial replacement of the cement volume and with corn cobs/sunflower stalks aggregates as partial replacement of mineral aggregates.

The density measured 28 days after casting of the composites with vegetal aggregates, without fly ash, decreased as the share of biomaterial increased. Lightweight concrete can be obtained with a percentage of more than 50% of vegetal matter.

In the case of using only vegetal aggregates in the composite recipe, the lowest density was obtained by the material with corn cobs. Sunflower stalks composites recorded bigger densities than the corn cobs ones.

Among the analyzed variants, the best results in terms of density-compressive strength rate were obtained in the cases of the composites with 50% sunflower aggregates and fly ash, no matter of the fly ash rates. A significant improvement effect was also obtained when was replaced a 20% of the cement volume with fly ash in the composite recipe with 25% aggregates of sunflower stalks.

From the splitting tensile strength perspective, a positive influence had the 10-replacement rate of the cement volume with fly ash in the compositions with 25 and 50% corn cobs aggregates. In the case of composite with 50% sunflower aggregates, fly ash led to improvements of this parameter even in the case of using a cement replacement rate of 30%.

Fly ash decreased the elasticity of the composites with 50% of sunflower aggregates.

Regarding the resistance to repeated freeze-thaw cycles, a positive influence on the performance of composites with vegetal aggregates had the replacement of 10% of the cement volume with fly ash in the composition with 50% corn cobs aggregates and the replacement with 10, 20, and 30% of the cement volume with fly ash in the compositions with 25 and 50% sunflower stalks aggregates.

The association of fly ash–corn cobs aggregates and fly ash–sunflower stalks aggregates led to unpredictable results, without preserving a certain logic path. For this reason, research must be continued in more detail, applying smaller steps in the replacement rates of the mineral aggregates mainly.

The idea of using plant aggregates and fly ash in making lightweight concrete is reasonable, ensuring a technical-economic efficiency worthy of consideration. The mechanical strengths related to the low specific weight, but, especially, the resistance to repeated freeze-thaw cycles, make them useful in prefabricated elements used in the external environment. If we consider the very large quantities of prefabricated elements used for roads (such as curbs, gutters, paving slabs, etc.) and irrigation systems (manholes, pipes, canals, etc.) and their replacement rate, it can be concluded that the use of bio-based construction composite is a solution of the future. The large quantities of concrete used in infrastructure of communication routes and irrigation systems, as well as the working environment, can create the premises for replacing ordinary concrete with the one developed in this research which can ensure a higher degree of impermeability, improved resistance to frost-thaw, and finally an increased durability and lower maintenance or replacement costs.

In the same vein, the lifetime period of buildings or various building elements plays a predominant role in assessing the impact on the environment by increasing carbon emissions due to their frequent replacement due to low durability. To this can be added a continuous and accentuated degradation of the environment due to the depletion of raw materials and conventional energy sources used in the extraction, production, and transport of construction materials, their implementation, as well as their deconstruction and reintegration into the environment.

As a result, finding new materials to ensure an increase in the durability of constructions as a whole or of buildings parts, while maintaining the physical-mechanical and chemical characteristics at an acceptable level compared to those of classical materials, is a current and perspective concern for all factors involved in the construction sector, and this research proposes just that.

## Figures and Tables

**Table 1:** Organic chemical composition of corn cobs and sunflower stalks.

Sample	DM	Humidity	100% DM	Gross Ash		Organic Substance		Gross Protein		Gross Fat		Gross Cellulose		NNE	
				DM	100% DM	DM	100% DM	DM	100% DM	DM	100% DM	DM	100% DM	DM	100% DM
Corn cobs	91.83	8.17	100	1.79	1.95	90.04	98.05	1.9	2.07	0.59	0.65	33.21	36.16	54.34	59.17
Sunflower stalks	91.57	8.43	100	8.79	9.60	82.78	90.40	4	4.37	0.54	0.6	48	52.41	30.24	33.02

**Table 2:** Particular elements of the recipes developed and analyzed in the study.

Crt. No.	Composite Recipe Symbol	Binder		Aggregates		
		Cement	Fly Ash	Mineral Aggregates	Corn Cobs Aggregates	Sunflower Stalks Aggregates
		[% of Binder]	[% of Binder]	[% of Aggregates]	[% of Aggregates]	[% of Aggregates]
1	RC	100	0	100	0	0
2	CF10	90	10	100	0	0
3	CF20	80	20	100	0	0
4	CF30	70	30	100	0	0
5	CC25	100	0	75	25	0
6	CC25-F10	90	10	75	25	0
7	CC25-F20	80	20	75	25	0
8	CC25-F30	70	30	75	25	0
9	CC50	100	0	50	50	0
10	CC50-F10	90	10	50	50	0
11	CC50-F20	80	20	50	50	0
12	CC50-F30	70	30	50	50	0
13	CS25	100	0	75	0	25
14	CS25-F10	90	10	75	0	25
15	CS25-F20	80	20	75	0	25
16	CS25-F30	70	30	75	0	25
17	CS50	100	0	50	0	50
18	CS50-F10	90	10	50	0	50
19	CS50-F20	80	20	50	0	50
20	CS50-F30	70	30	50	0	50

**Table 3:** Classification of cement-based composites developed in this research.

Density Mass Class	Density Mass Range [Kg/M^3^]	Cement-Based Composites
D1.8	(1600, 1800]	CC50, CS50 CC50-F10, CC50-F20, CC50-F30 CS50-F10, CS50-F20, CS50-F30
D2.0	(1800, 2000]	CC25-F10, CC25-F20, CC25-F30 CS25-F10, CS25-F20, CS25-F30

**Table 4:** Strength classes of the studied lightweight aggregate concretes.

Strength Classes of Lightweight Aggregate Concrete	Cement-Based Composites
LC8/9	CC25, CS25, CC25-F10 CS25-F10, CS25-F30, CS50-F10, CS50-F20
LC12/13	CS25-F20

## Data Availability

Data is contained within the article and in [31] for the method of vegetal aggregates production.
